# CtBP2 proteome: Role of CtBP in E2F7-mediated repression and cell proliferation

**DOI:** 10.18632/genesandcancer.2

**Published:** 2014-01

**Authors:** Ling-Jun Zhao, T. Subramanian, S. Vijayalingam, G. Chinnadurai

**Affiliations:** ^1^ Institute for Molecular Virology Saint Louis University Health Sciences Center Doisy Research Center 1100 South Grand Blvd St. Louis, Missouri 63104

**Keywords:** CtBP2, Proteome, E2F7, E2F1, NuRD

## Abstract

C-terminal binding protein (CtBP) family transcriptional corepressors include CtBP1 and CtBP2. While CtBP1 and CtBP2 share significant amino acid sequence homology, CtBP2 possesses a unique N-terminal domain that is modified by acetylation and contributes to exclusive nuclear localization. Although CtBP1 and CtBP2 are functionally redundant for certain activities during vertebrate development, they also perform unique functions. Previous studies have identified several CtBP1-interacting proteins that included other transcriptional corepressors, DNA-binding repressors and histone modifying enzymatic components such as the histone deacetylases and the histone demethylase LSD-1. Here, we carried out an unbiased proteomic analysis of CtBP2-associated proteins and discovered the association of several components of the CtBP1 proteome as well as novel interactions. The CtBP2 proteome contained components of the NuRD complex and the E2F family member E2F7. E2F7 interacted with the hydrophobic cleft region of CtBP1 and CtBP2 through a prototypical CtBP binding motif, PIDLS. E2F7 repressed E2F1 transcription, inhibited cell proliferation in a CtBP-dependent fashion. Our study identified CtBP as a corepressor of E2F7 and as a regulator of DNA damage response.

## INTRODUCTION

CtBP 1 and CtBP2 are highly related transcriptional regulators and are implicated in a multitude of cellular functions. They (collectively referred here as CtBP) function predominantly as transcriptional co-repressors, in addition to certain diverse cytosolic functions (reviewed in [1, 2]). In addition to transcriptional repression, invertebrate and vertebrate CtBPs also function as context-dependent transcriptional activators [3-5]. While CtBP1 is localized both in the cytosol and nucleus, CtBP2 is nuclear due to the presence of a unique 20-amino acid N-terminal domain (NTR). The function of CtBP2 NTR is regulated by acetylation by p300 [6]. As inferred from their amino acid sequence homology and similarities in three dimensional structures [7, 8](Pilka, ES et al., MMDB IP:4438), CtBP1 and CtBP2 are functionally redundant in the regulation of gene expression during animal development. However, they also perform unique developmental functions. Mice deficient in CtBP1 were viable, albeit, with reduced lifespan while CtBP2 null mice exhibited developmental defects beyond E10.5 and were not viable [9]. Differential interaction of CtBP cofactors may contribute to the functional difference between CtBP1 and CtBP2.

Between CtBP1 and CtBP2, the interacting proteins of CtBP1 have been more extensively characterized. A proteomic analysis of CtBP1-associated proteins revealed interaction with DNA binding repressors such as ZEB, corepressors such as CoREST and Znf217, class I histone decetylases 1 and 2 and the histone demethylase LSD-1 [10]. Mutational analysis of the interaction of different CtBP1-interacting proteins led to a model that the CtBP1 dimer may interact with promoter-bound repressors with one of the two hydrophobic clefts of the dimer while the other hydrophobic cleft region may interact with various histone modifying enzymes either directly or through other corepressors [11]. In addition to the proteomic analysis, other protein interaction studies have also identified other CtBP1-binding proteins under various contexts. In contrast to CtBP1, only limited attempts have been made to identify CtBP2-interacting proteins which resulted in the identification of proteins such as the tumor suppressor proteins HDM2/MDM2 [12] and ARF [13]. Here, we have carried out an unbiased proteomic study to identify CtBP2-associated proteins. Our analysis has identified several novel CtBP2-interacting proteins which include E2F7 and components of the nuclear remodeling histone deacetylase (NuRD) complex [14]. We demonstrate that both CtBP1 and CtBP2 interact with E2F7 and play critical roles during E2F7-mediated repression of E2F1 and cell proliferation. We also provide evidence that CtBP2 preferentially interacts with p66-beta subunit of the NuRD complex in a manner dependent on the NTR of CtBP2.

## RESULTS

### CtBP2-interacting proteins

To examine whether CtBP2 interacts with unique cellular factors, we generated a HeLa cell line stably expressing Flag-HA-tagged CtBP2 (FH-CtBP2). FH-CtBP2 cells were either untreated or treated with TSA, an inhibitor of histone deacetylases, before cell lysates were prepared for purification of CtBP2-bound proteins by Flag and HA double affinity purification. The FH-CtBP2 protein complex was subjected to LC-MS analysis. Comparison of TSA-treated and -untreated samples revealed subtle quantitative differences in certain CtBP2-bound proteins. However, both preparations of FH-CtBP2 bound to the same set of proteins. These proteins included previously identified proteins that bind to CtBP1 (reviewed in [2]), as well as novel proteins (Table 1). Among the novel proteins, E2F7 and components of the Nucleosome Remodeling Deacetylase (NuRD) complex, including CHD4 and p66-beta (reviewed in [15], have recently been shown to play critical roles in cell cycle regulation and chromatin histone modifications. Interestingly, a candidate PIDLS CtBP-interaction motif is present close to the N-terminus of E2F7, and a PLDLS-like motif PVDMS is also located in the NuRD component p66-beta. Potential PLDLS-like motifs are also present in the Zinc finger protein TRPS1 and the WD repeat containing protein C2ORF44; however, the cellular functions of these proteins remain to be examined.

**Table 1:** List of CtBP2-bound proteins Numbers displayed for the cell lines are number of unique peptides identified.

CtBP2 binding proteins (previously identified for CtBP1)	Accession Number	Molecular Weight	HeLa	CtBP2	CtBP2 (+TSA)
C-terminal-binding protein 2 isoform 1	gi|4557499	49 kDa	2	42	48
Zinc finger protein 217	gi|5730124	115 kDa	0	32	47
C-terminal-binding protein 1 isoform 1	gi|4557497	48 kDa	2	19	21
Amine oxidase (flavin containing) domain 2, isoform CRA_a	gi|119615437	100 kDa	0	15	24
Zinc finger E-box-binding homeobox 1 isoform a	gi|189409130	124 kDa	0	14	14
Adenomatous polyposis coli	gi|182397	312 kDa	0	11	13
Histone deacetylase 1-like isoform 6	gi|13138860	55 kDa	0	10	13
Histone deacetylase 2-like isoform 2	gi|293336691	55 kDa	0	8	7
Ligand-dependent corepressor isoform 1	gi|282847504	47 kDa	0	12	11
REST corepressor 1	gi|344925845	53 kDa	0	3	11
Ligand dependent nuclear receptor corepressor-like isoform 1	gi|260764001	67 kDa	0	3	5
					
**Novel CtBP2 binding proteins**					
Transcription factor E2F7	gi|145580626	100 kDa	0	4	4
Mi-2 protein (CHD4)	gi|1107696	218 kDa	0	13	16
Transcriptional repressor p66-beta	gi|21218438	65 kDa	0	11	10
Methyl-CpG binding domain protein 3, isoform CRA_b	gi|119589885	26 kDa	0	3	5
Metastasis-associated gene	gi|1008544	81 kDa	0	5	3
Metastasis-associated protein MTA2	gi|14141170	75 kDa	0	11	14
Metastasis associated 1 family, member 3, isoform CRA_d	gi|119620722	59 kDa	0	3	3
Retinoblastoma binding protein 7, isoform CRA_b	gi|119619326	52 kDa	0	4	2
Zinc finger transcription factor TRPS1	gi|6684534	142 kDa	0	8	10
WD repeat-containing protein C2orf44 isoform 1	gi|13376798	79 kDa	0	6	7
Chromosome 20 open reading frame 112, isoform CRA_b	gi|119596774	64 kDa	0	6	6
Human Dead-Box Rna Helicase Ddx3x	gi|114794734	47 kDa	0	2	2
Rho GTPase-activating protein 21	gi|203097003	217 kDa	0	1	2

### Specific interaction of CtBP2 with E2F7 and NuRD components

**Figure 1:**
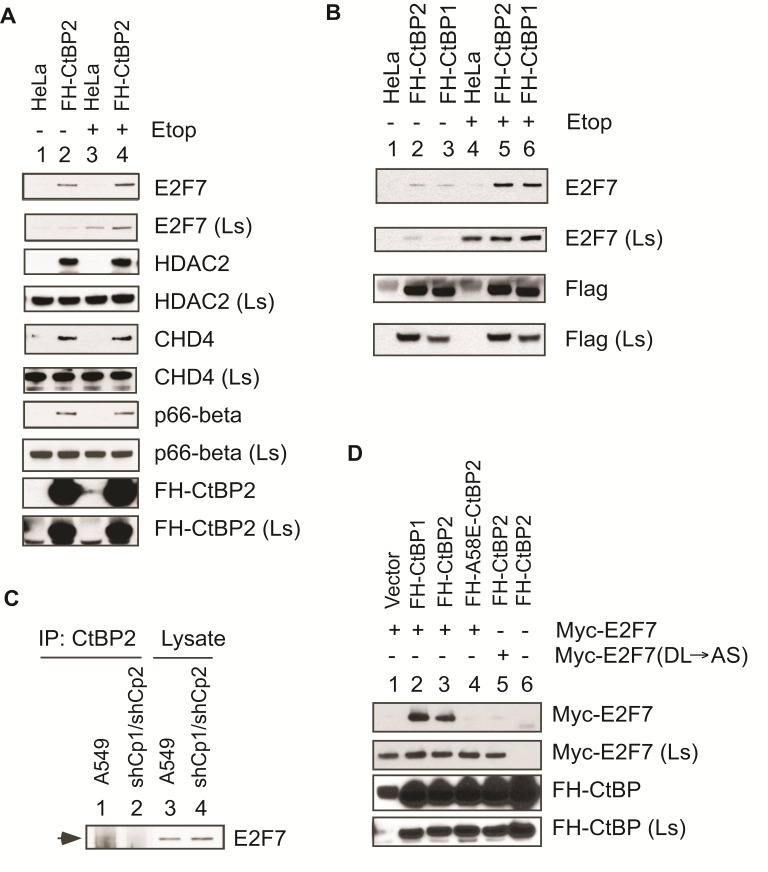
CtBP interaction with E2F7 and other proteins A. HeLa and HeLa/FH-CtBP2 cell line (cell line #1) which stably expresses Flag-HA-tagged CtBP2 were untreated or treated with 10 μM etoposide for 6 hr, and then analyzed for binding of FH-CtBP2 to E2F7 and other cellular proteins by coimmunoprecipitation of cell lysates with Flag antibody beads followed by Western blot analyses with indicated antibodies. Ls: Cell lysate.B. FH-CtBP2 (cell line #2), FH-CtBP1 and HeLa cells were treated with etoposide and immunoprecipitation and Western blot analyses were carried out as in A. C. A549 cells and CtBP1/2 down-regulated A549 cells were lysed and immunoprecipitated with CtBP2 antibody and analyzed by Western blotting using E2F7 antibody. D. FH-tagged CtBP constructs and Myc-E2F7 constructs were co-transfected into HeLa cells in combinations as indicated, and the interaction between CtBP and Myc-E2F7 proteins was examined as in A, except that 2 μg of a non-specific IgG was used during co-immunoprecipitation with the Flag antibody beads to reduce non-specific binding of Myc-E2F7 to the Flag antibody beads. Ls: Cell lysate.

To confirm the interaction between CtBP2 and the identified components of the CtBP2 protein complex, lysates from the FH-CtBP2 cell line and normal HeLa cells were used for co-immunoprecipitation with the Flag antibody. Western blots of the precipitated proteins were performed with antibodies as indicated (Fig.1A, lanes 1 and 2). Since E2F7 expression is subject to regulation by DNA damage response [16-18], we analyzed the same set of proteins in cells treated with etoposide (Fig.1A, lanes 3 and 4). As shown, in HeLa cells E2F7 bound to the Flag antibody to some background levels (lane 1); however, in cells expressing FH-CtBP2 the bound E2F7 signal was much stronger (lane 2). Etoposide treatment enhanced the level of E2F7 expression in both HeLa and FH-CtBP2 cell lines (compare lanes 3 and 4 with lanes 1 and 2). Correspondingly, an increased amount of E2F7 was associated with FH-CtBP2 (lane 4). By this analysis, CtBP2 was also shown to interact with the NuRD components, HDAC2, CHD4 and p66-beta. Factors such as ZNF217/CoREST/LSD-1 which were identified in the CtBP1 proteome [10] also readily interacted with CtBP2 (data not shown). However, the interaction of these factors with CtBP2 did not seem to be significantly influenced by etoposide treatment.

To substantiate the interaction between CtBP2 and E2F7, we examined a separate clone of HeLa/FH-CtBP2 and a HeLa/FH-CtBP1 clone (Fig.1B) by a similar approach. The results showed that both CtBP2 and CtBP1 interacted with E2F7 specifically (Fig.1B, top panel) since the control HeLa cells were negative for the assay (lanes 1 and 4). To ascertain if endogenous CtBP2 interacts with E2F7, normal A549 cells and A549 cells with CtBP1 & 2 double knock-down were used to prepare lysates, which were then immunoprecipitated with a CtBP2 antibody (Fig.1C, lanes 1 and 2). As shown, CtBP2 antibody pulled out E2F7 only from the normal A549 cells (lane 1) but not from the CtBP1 & 2 knock-down cells (see Fig.4A for CtBP1&2 Western blots), suggesting that endogenous CtBP2 and E2F7 interact with each other.

### E2F7 interacts with CtBP through a canonical CtBP binding motif

CtBP interaction with E2F7 was examined further by co-transfection of FH-CtBP expression constructs together with the Myc-tagged E2F7 expression construct (mouse E2F7 clone; [19] (Fig.1D). Cell lysates were co-immunoprecipitated with the Flag antibody and the precipitated proteins were examined by western blot analysis. In this analysis, both CtBP1 and CtBP2, as well as the CtBP2 mutant A58E [mutation located within the hydrophobic region that binds to PLDLS-like motifs [20]] were examined. As shown, both CtBP1 (lane 2) and CtBP2 (lane 3) bound to Myc-E2F7, and yet the A58E-CtBP2 mutant did not (lane 4), suggesting that CtBP interaction with E2F7 requires a PLDLS-like motif. Visual examination of the E2F7 sequence revealed the presence of a canonical CtBP-binding motif, PIDLS at the N-terminal region. To examine the role of this motif in E2F7 interaction with CtBP2, we generated a Myc-E2F7 mutant with DL→AS mutation within the PIDLS motif. A similar mutation within the prototypical CtBP-binding protein, adenovirus E1A has been shown to abolish CtBP interaction [21, 22]. This mutant failed to interact with CtBP2 (Fig. 1D, lane 5). These results suggest that E2F7 interacts with CtBP2 through a canonical CtBP-binding motif with the hydrophobic cleft region of CtBP2.

### Unique interaction of p66-beta of the NuRD complex with CtBP2

**Figure 2:**
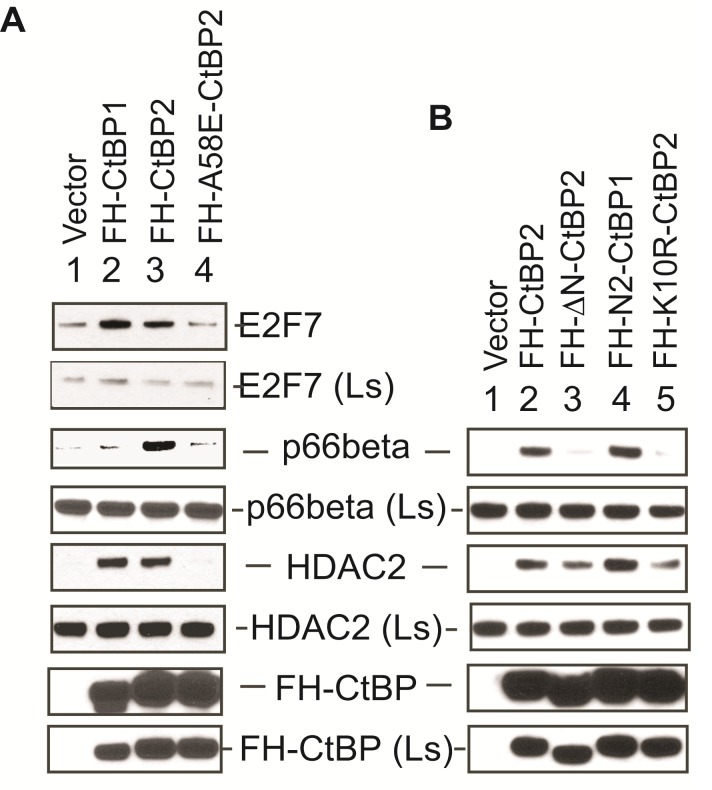
Unique interaction of CtBP2 with p66-beta A. Role of CtBP2 hydrophobic cleft. B. Role of CtBP2 NTR. Flag-HA-tagged CtBP expression constructs were transfected into HeLa cells, and the interaction of the transiently expressed CtBP proteins with cellular proteins was analyzed as in Fig. 1. Ls: Cell lysate.

In an attempt to determine whether CtBP1 and CtBP2 interact with any identified proteins differently, we transfected Flag-HA-tagged CtBP1 and CtBP2 expression constructs into HeLa cells and examined their interaction with different proteins. As shown in Fig. 2A, both CtBP1 and CtBP2 interacted with endogenous E2F7 and HDAC2. In contrast, the interaction with p66-beta was detected only with CtBP2 (lane 3). In addition, the A58E-CtBP2 mutant, which does not interact with PLDLS-like motifs, did not interact with any of these proteins. Thus, interaction with the NuRD component, p66-beta is unique to CtBP2 and appears to interact with the hydrophobic cleft region of CtBP2.

Since both CtBP1 and CtBP2 interacted with majority of partner proteins through PLDLS-like motifs and yet CtBP1 did not bind to p66-beta, we wondered whether the unique sequence in the CtBP2 N-terminal region (NTR) contributes to this specificity. The CtBP2 mutant ΔN-CtBP2 which lacks the NTR, and N2-CtBP1 mutant in which the CtBP1 N-terminus is modified to contain CtBP2 NTR, were then transfected into HeLa cells and their interaction with co-factors was examined. As shown in Fig. 2B, deletion of the NTR from CtBP2 abolished CtBP2 interaction with p66-beta (lane 3) while maintaining CtBP2 interaction with HDAC2. Interestingly, acquisition of CtBP2 NTR rendered CtBP1 capable of interaction with p66-beta (lane 4). Thus, CtBP2 interaction with p66-beta appears to require both the NTR and the PLDLS-binding cleft of CtBP2.

We previously demonstrated that the unique CtBP2 NTR renders CtBP2 capable of nuclear localization as well as the ability to be acetylated [6]. One of the Lys residues within the NTR, Lys10, appeared to be critical for acetylation as well as CtBP2 nuclear localization. To examine if this residue is also critical for CtBP2 interaction with p66-beta, we transfected HeLa cells with the K10R-CtBP2 mutant and found that K10R mutation abolished CtBP2 interaction with p66-beta (Fig. 2B, lane 5). Thus, both CtBP2 nuclear localization and ability to interact with p66-beta require Lys10 residue of CtBP2.

### Role of CtBP in E2F7-dependent repression of E2F1

**Figure 3:**
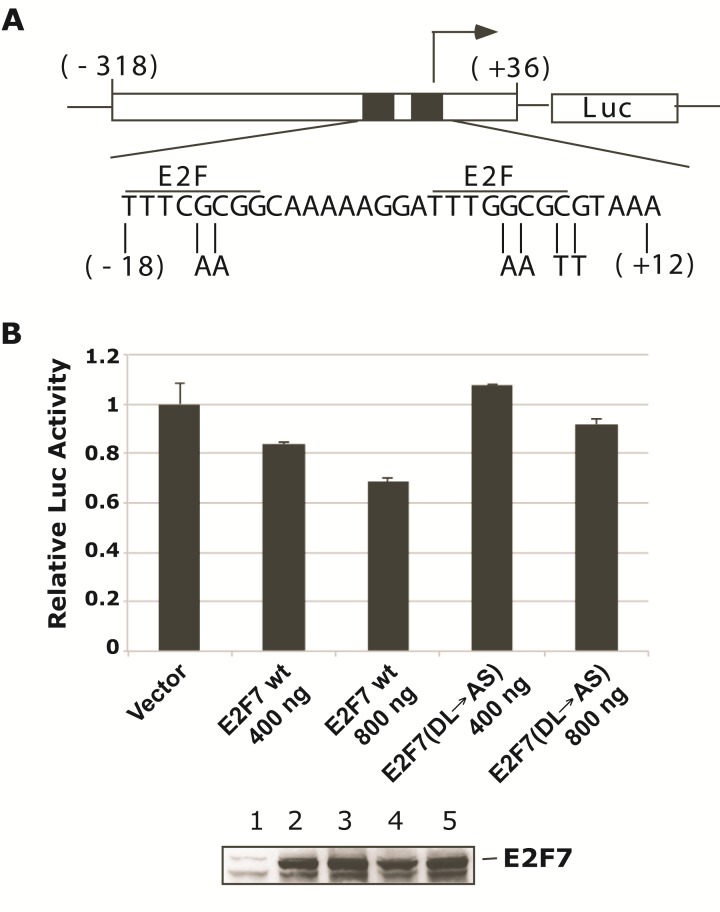
E2F1 promoter regulation by CtBP A. Diagram of E2F1-Luc and E2F1-mu-Luc constructs. The E2F1 promoter region from (−)318 to (+)36 was PCR amplified and cloned into the pG5-Luc vector. The two E2F motifs in this promoter region were mutated as indicated at the bottom. B. E2F1-Luc was co-transfected with Myc-E2F7 or Myc-E2F7(DL→AS) mutant at indicated amounts, and Luc activity was determined. Only Luc data was plotted since Myc-E2F7 caused a severe repression of hRL-tk internal control. The expression of wt and mutant E2F7 proteins is shown at the bottom.

E2F7 has recently been identified as a p53-responsive E2F family member that is activated during DNA damage response [17, 18]. E2F7 has also been identified as the repressor that regulates E2F1 expression [16-18]. Therefore, it is logical to expect that CtBP may serve as the corepressor of E2F7 to repress E2F1. To determine the role of CtBP in E2F7-dependent repression of E2F1, first we carried out a luciferase reporter assay. For this purpose, we generated a luciferase reporter construct which contains an approximately 300-bp promoter region of E2F1 (Fig. 3A), including the two E2F binding sites. HeLa cells were transfected with the E2F1-Luc reporter and E2F7 wt or E2F7 (DL→AS) mutant and the luciferase activity was determined (Fig. 3B). Transfection of E2F7 wt efficiently repressed the E2F1 promoter activity. In contrast, E2F1 promoter repression by E2F7 was significantly reduced by the DL→AS mutation.

**Figure 4:**
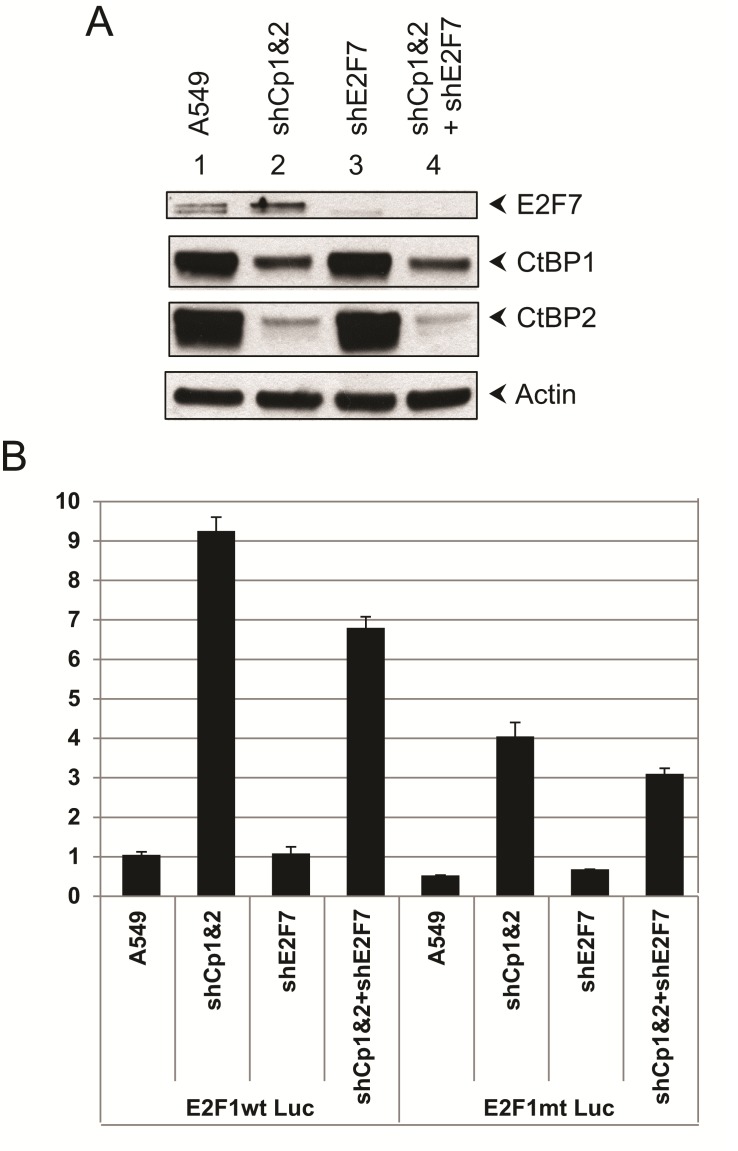
Effect of CtBP-depletion and etoposide treatment on E2F1 promoter activity A. Protein expression profiles in A549 cells depleted for CtBP1/2 (shCp1&2) and cells depleted for CtBP1/2 and E2F7 (shE2F7). Five days after lentiviral infection, cells were lysed and examined by Western blots. B. E2F1-wt-Luc and E2F1-mu-Luc constructs were transfected into A549, shCtBP1&2, shCtBP1/2+shE2F7 cell lines. Cells were lysed and luciferase assays performed. Experiment was performed in triplicates, and standard deviations are shown.

We also determined the effects of endogenous CtBP1 and CtBP2 on the E2F1 promoter activity by using A549 cells that were acutely depleted of both CtBP1 and CtBP2 by infection with lentiviral vectors that express shRNAs against CtBP1 and CtBP2 followed by short term drug selection. As shown in Fig. 4A, CtBP1 and CtBP2 shRNAs caused efficient reduction in the expression of CtBP1 and CtBP2 levels (Lane 2). Similarly, we also generated shE2F7 (Fig. 4A, lane 3) and shE2F7/shCtBP1&2 triple knock-down cells (Fig. 4A, lane 4). The reduction in the levels of the intended target proteins was as expected as shown. Subsequently, E2F1wt-Luc or E2F1mt-Luc construct was transfected into normal A549 cells or the shRNA cell lines, and luciferase assays were performed to evaluate E2F1 promoter activity. As shown in Fig. 4B, knock-down of CtBP1&2 enhanced E2F1 promoter activity, suggesting that CtBP1&2 are important for repression of E2F1 promoter. In contrast, knock-down of E2F7 alone did not have a significant impact. This result suggests that in the absence of E2F7, there may be alternative mechanisms to repress the E2F1 promoter. Alternatively, the cells might need to readjust to a reduced E2F7 level and recruit alternative repressors to regulate the E2F1 promoter. When E2F7 and CtBP1/2 were all knocked down, E2F1 promoter activity was also enhanced compared to normal A549 cells. However, the extent of the increase was not as high as when CtBP1&2 were knocked down. Thus, in the absence of CtBP1 and 2, E2F7 might have lost its ability to repress E2F1 promoter. When the E2F1mt-Luc reporter DNA was used for transfection, the Luc activity was lower compared to the E2F1wt-Luc, suggesting that the observed effects of shRNAs on E2F1 promoter were most likely exerted through the two E2F elements in the E2F1 promoter, which are also the targets of E2F7.

**Figure 5:**
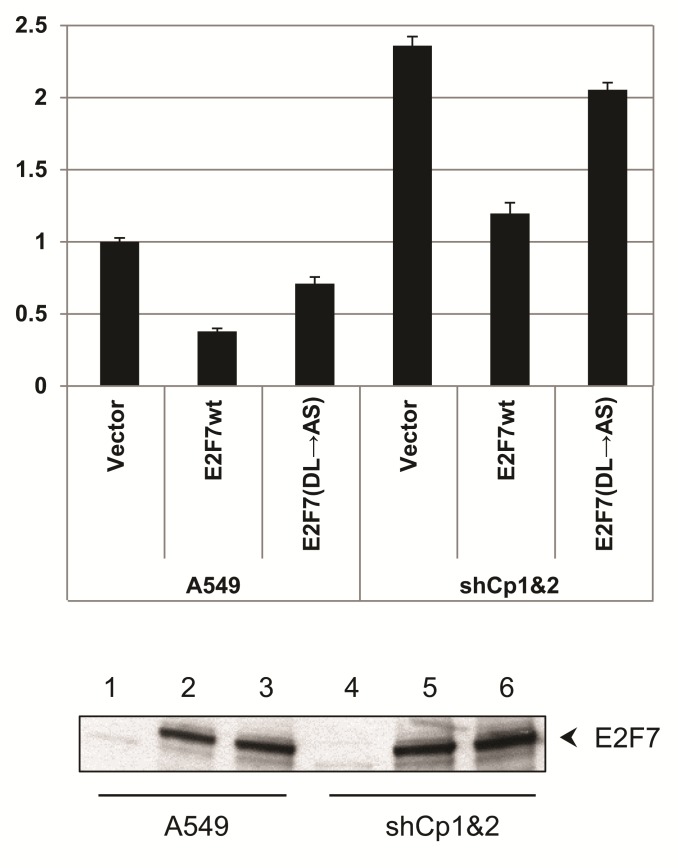
Effect of E2F7 overexpression on E2F1 promoter in CtBP-depleted cells E2F1 promoter activity in CtBP1/2-depleted cells. Cells were co-transfected with E2F1-Luc, hRL-tk and 800 ng of Myc-E2F7 or Myc-E2F7(DL→AS), and the luciferase assays performed. Bottom: Western blot for Myc-E2F7 with Myc antibody.

The observation that CtBP1&2 knock-down cells had a higher level of E2F1 promoter activity was consistent with CtBP1/2 being co-repressors for E2F7. To examine this possibility further, we co-transfected normal A549 cells and the CtBP1/2 double knock-down cells with E2Fwt-Luc and E2F7 wt or E2F7(DL-AS). As expected, luciferase assays showed repression of E2F1-Luc in normal A549 cells by E2F7wt and much less efficiently by E2F7(DL-AS) (Fig. 5). In CtBP1&2 double knock-down cells, over-expression of E2F7wt or E2F7(DL-AS) mutant still repressed the E2F1 promoter. This result could be partially due to the low level of CtBP1&2 expression in the CtBP1/2 double knock-down cells. Alternatively, E2F7 could have additional mechanisms to repress E2F1 promoter in the absence of CtBP1/2.

### Effect of CtBP-E2F7 interaction on cell proliferation and cell cycle

**Figure 6:**
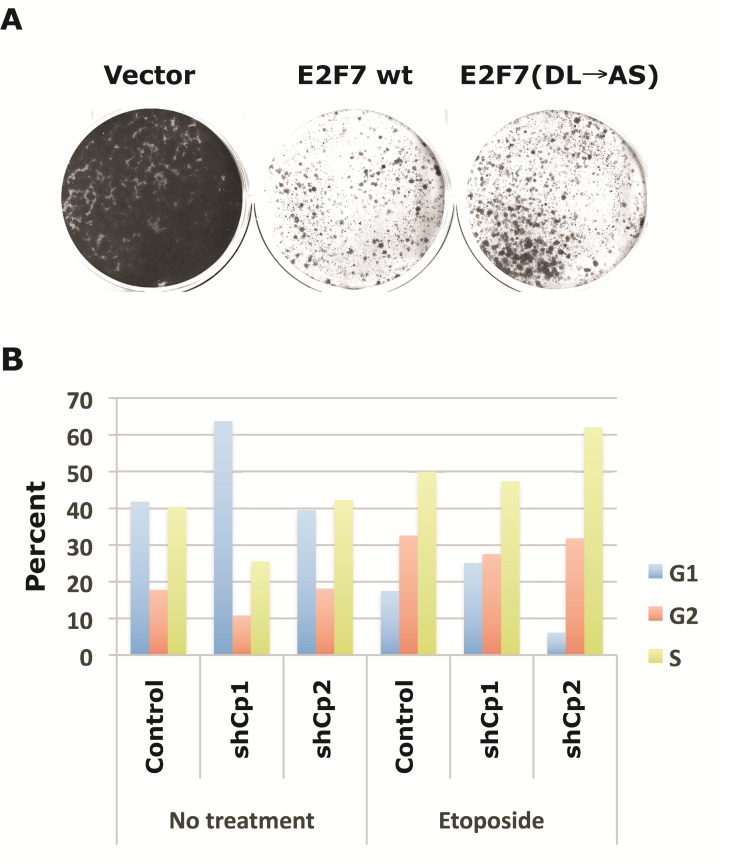
Effect of E2F7 and CtBP2 on cell proliferation and cell cycle regulation A. Effect of E2F7 on cell proliferation. U2OS cells were transfected with vector, E2F7 wt or E2F7(DL→AS), and selected with 400 μg/ml of G418 for 2 weeks. Drug-resistant cells were stained with crystal violet after two weeks. B. Effect of CtBP2 depletion and etoposide on cell cycle. Cells depleted for CtBP1 (shCp1) or CtBP2 (shCp2) were either untreated or treated with etoposide (10 μM) for 16 hr, and processed for cell cycle analysis.

To examine the functional significance of CtBP-E2F7 interaction, we determined the effect of E2F7 wt and E2F7 (DL→AS) (defective in CtBP interaction) mutant on proliferation of U2OS cells (Fig. 6A). Transfection of E2F7 wt into U2OS cells resulted in substantial reduction in colony formation while the E2F7(DL→AS) mutant generated more and bigger colonies (compared to cells transfected with E2F7 wt). These results suggest that the interaction of CtBP with E2F7 may contribute to the inhibition of cell proliferation.

Since E2F7 modulates DNA damage response through regulation of genes such as E2F1, U2OS cells with CtBP1 or CtBP2 knock-down were also used to determine the effect of CtBP on cell cycle regulation in response to treatment with etoposide (Fig. 6B). When subjected to cell cycle analysis, untreated CtBP1 knock-down cells appeared to have a G1 cell cycle block, while CtBP2 knock-down cells seemed to be similar to the control U2OS cells. After etoposide treatment, however, CtBP2 knock-down cells appeared to have an accelerated G1-S transition, as is indicated by the higher proportion of cells at S phase and the lower proportion of cells at G1. In contrast, most of the CtBP1 knock-down-induced changes in untreated cells and disappeared after etoposide treatment. Thus, knock-down of CtBP1 and CtBP2 appeared to affect the cell cycle differently, with CtBP2 knock-down exerting a more significant impact upon etoposide treatment.

## DISCUSSION

By proteomic analysis, CtBP2 was found to interact with several new proteins in addition to certain previously identified proteins that bind to CtBP1 (reviewed in [2]) (Table 1). In the current study, we focused on E2F7 since it is the effector molecule of p53-mediated transcriptional repression and plays important roles in the expression of the critical trans-activating E2F family member, E2F1 that regulates cell proliferation and apoptosis [17, 18]. Although we identified E2F7 in the CtBP2-protein complex, we showed that it interacts with CtBP1 as well. During the preparation of this manuscript another report identified CtBP1 and CtBP2 as E2F7-interacting proteins [23]. Our results indicate that E2F7 interacts with the hydrophobic cleft region of CtBP since a mutation of a critical residue A58 of CtBP2 (corresponding to A52 of CtBP1) within the hydrophobic cleft region abolished E2F7 interaction. We also identified a prototypical CtBP binding motif PIDLS within the N-terminal region of E2F7 that mediates interaction with CtBP. Although presence of other CtBP-binding sequence elements within E2F7 cannot be ruled out, the DL→AS mutation within the core PIDLS motif abolished interaction with CtBP suggesting that this motif may be the primary binding sequence. We note that although the DL→AS mutation in E2F7 effectively abolished E2F7 interaction with CtBP (Fig. 1B), it only partially relieved the repressive activity of E2F7 in the transient reporter assay (Fig. 3) or E2F7-mediated inhibition of cell proliferation (Fig. 6A). These results suggest E2F7 may recruit other co-repressor(s), in addition to CtBP. They also raise the possibility that selective inactivation of CtBP functions may relieve the repressive action of E2F7 which could be exploited in chemotherapy regimens (see below).

Although E2F7 has been identified as a negative regulator of E2F1 and several other proliferation genes [18], the mechanism of transcriptional repression by E2F7 was not known. Our results suggest that CtBP may be a corepressor of E2F7. Thus, CtBP appears to be an important regulator of the E2F7-E2F1 regulatory loop that is activated by p53 during DNA damage and regulate cell proliferation and apoptosis. Since CtBP is overexpressed in several cancers [17, 24-30] targeting CtBP may enhance the chemotherapeutic effects of agents that activate p53 and E2F7. E2F7 overexpression has been identified as a prominent hall mark of squamous cell carcinomas (SCC) [31, 32]. Since the relative chemoresistance of these carcinomas appears to be linked to high E2F7/E2F1 ratio, targeting CtBPs may enhance the expression of E2F1 and the apoptotic response. Since molecules that selectively disrupt CtBP functions are emerging [33, 34], such molecules may be exploited for the treatment of SCC.

Here, we have also identified different components of the NuRD complex in CtBP2 proteome. One of these components, p66-beta interacted with CtBP2 in a manner dependent on the N-terminal region (NTR) of CtBP2 suggesting that NuRD may be a CtBP2-specific repression effector. We have previously shown that acetylation of K10 within the NTR is essential for nuclear targeting of CtBP2 [6]. The interaction of p66-beta was also dependent of K10 (Fig. 2B). It is possible that CtBP2 NTR may play a more direct role in interaction with p66-beta other than nuclear targeting of CtBP2. Although a fraction of CtBP1 is normally localized in the nucleus, we did not detect significant interaction of p66-beta with nuclear localized CtBP1 by immunofluoresence analysis (not shown). Interestingly, in addition to the NTR, the interaction of p66-beta was also dependent on the hydrophobic cleft region since CtBP2 A58E mutant was deficient in interaction. We note that p66-beta contains a putative CtBP-binding motif PVDMS. Since the interaction of p66-beta with CtBP2 was generally stronger in co-immunoprecipitation experiments while the interaction of other NuRD subunits was variable, we believe that p66-beta may the linker that connects NuRD with CtBP2. It is noteworthy that the activities of the NuRD complex are also linked to cancer, DNA replication and in DNA repair [35, 36]. Future studies will determine the contexts in which CtBP2 may recruit the NuRD complex to regulate chromatin modifications and gene expression.

## MATERIALS AND METHODS

### Proteomic analysis

HeLa cells were transfected with pFH-CtBP2 plasmid [6] for 24 hr, trypsinized and plated at a low density in 6-well plates. After selection with G418 (400 μg/ml), well separated single cell colonies were cloned and expanded. Expression of FH-CtBP2 in the cell line was confirmed by Western blots as well as immunofluorescence analysis with Flag-Cy3 antibody. For proteomic analysis of CtBP2-interacting proteins, whole cell lysates from fifteen 100 mm dishes of HeLa/FH-CtBP2 cells were immunoprecipitated the Flag antibody and the bound proteins were eluted with the Flag peptide, followed by a second step of purification with the HA antibody. Protein complexes bound to the HA antibody beads were directly subjected to trypsin digestion and proteomic analysis as described [37].

### DNA transfection for transient protein expression, lentivirus generation, and luciferase assays

For co-immunoprecipitation studies, HeLa cells were plated in 100 mm dishes at 1.6 × 10^6^ cells per dish the day before transfection. HeLa, U2OS, and A549 cells were usually plated at 5 × 10^4^ cells/well in 12-well plates for transfection to perform luciferase assays. All transfections were carried out with the XTreme HP transfection reagent (Roche) or Turbofect (Thermoscientific). To generate lentiviruses, 293T cells were plated in 6-well plates at 6×10^5^ cells/well, and transfected the next day with a DNA mixture of 1.2 μg shRNA plasmid (shCtBP1 or shCtBP2 plasmid,[38]), 1.2 μg of pCMV-Δ8.2ΔVpr (packaging DNA,[39]), and 0.6 μg of pCMV-VSV-G. After transfection, cells were maintained for 2 days, and the culture supernatant (2 ml) was filtered into 4 ml DMEM supplemented with 10% FBS and polybrene (8 μg/ml final concentration), and used to infect cells in a T75 flask with 4 × 10^5^ cells plated one day before infection. Selection of lentiviral infected cells (U2OS and A549 cells) was with 600 μg/ml of hygromycin B for shCtBP2, 6 μg/ml blasticidin for shCtBP1, or a combination of both drugs at 50% strength for dual selection of shCtBP1- and shCtBP2-infected cells. E2F7 shRNA cells were selected with 1 μg/ml of puromycin.

### Immunoprecipitation and western blot analysis

HeLa cells grown in 100 mm dishes were collected and lysed by freezing-thawing three times in 200 μl of a lysis buffer containing 20 mM Tris-HCl (pH8.0), 0.3 M KCl, 0.5 mM EDTA, 10 mM MgCl2, 10% glycerol, 0.1% NP40, 0.1% Tween-20, 0.5 mM DTT, 0.2 mM PMSF, and Protease Inhibitor Complete Mini (Roche). After rotating at 4°C for 30 min, cell debris was removed by centrifugation at 4°C for 5 min. The clear cell lysate was diluted with 20 mM Tris-HCl (pH8.0), and protein aggregates were removed again by centrifugation. The cell lysate was then mixed with 25 μl of Flag-agarose beads (Sigma), and incubated at 4°C for 1 hr. In the case of FH-CtBP co-transfection with Myc-E2F7, before incubation with the Flag antibody beads, the cell lysate was first pre-incubated with 2 μg of a mouse monoclonal antibody for luciferase (SCBT) to reduce non-specific binding of Myc-E2F7 to the Flag antibody beads. A 5 min centrifugation was done to remove potential protein aggregates. The antibody beads were washed twice with the cell lysis buffer diluted to 50% with 20 mM Tris-HCl (pH8.0). Bound proteins were eluted with a 2X SDS sample loading buffer without reducing agent for 10 min at room temperature. DTT (100 mM) was added to the eluted proteins before loading to a 4-12% gradient gel (Life Technologies) for Western blot analysis.

### Cell cycle analysis

U2OS Cells were trypsinized, pelleted, washed with PBS, and fixed with 70% ethanol. After pelleting and washing with PBS, cells were stained for 30 min with a propidium iodide (PI) solution (20 μg/ml PI, 0.2 mg/ml RNaseA, 0.1% Triton X-100 in PBS), and subjected to cell cycle analysis on FACSCalibur.
